# Perceived Severity and Susceptibility towards Leptospirosis Infection in Malaysia

**DOI:** 10.3390/ijerph17176362

**Published:** 2020-09-01

**Authors:** Surianti Sukeri, Wan Mohd Zahiruddin, Mohd Nazri Shafei, Rukman Awang Hamat, Malina Osman, Tengku Zetty Maztura Tengku Jamaluddin, Aziah Binti Daud

**Affiliations:** 1 Department of Community Medicine, School of Medical Sciences, Universiti Sains Malaysia, Kubang Kerian, Kota Bharu 16150, Kelantan, Malaysia; drzahir@usm.my (W.M.Z.); drnazri@usm.my (M.N.S.); aziahkb@usm.my (A.B.D.); 2 Department of Medical Microbiology and Parasitology, Faculty of Medicine, Universiti Putra Malaysia, Serdang 43400, Selangor, Malaysia; rukman@upm.edu.my (R.A.H.); malinaosman@upm.edu.my (M.O.); tengkuzetty@upm.edu.my (T.Z.M.T.J.)

**Keywords:** perceived severity, perceived susceptibility, perceived threat, leptospirosis, Health Belief Model, qualitative, Malaysia

## Abstract

Background: Perceived severity and susceptibility refers to one’s belief of the seriousness and the risk of contracting a specific disease. It is an essential study in public health as it assists in the understanding of the motivating factors towards disease prevention. This qualitative study aimed to explore perceived severity and susceptibility towards leptospirosis infection among respondents in two states of Malaysia. Methods: Focus group discussions using the phenomenology approach was conducted involving 72 respondents in Selangor and Kelantan. Data were examined using content analysis. Results: Respondents perceived leptospirosis infection as severe due to its poor disease prognosis and complications. However, some rated it less severe when compared with other chronic diseases such as cancer and AIDS. Their perceptions were influenced by their knowledge about the disease, media portrayal and frequency of health campaigns by the government. All respondents believed they were not susceptible to the disease. Conclusion: The low perceived susceptibility of leptospirosis infection is a matter of concern as it may contribute to respondents’ lack of motivation towards preventing the disease. The study findings may provide the basis for health promotional activities designed to heighten public perceived threat towards leptospirosis infection and thereby improving preventive health behaviors for avoiding leptospirosis.

## 1. Introduction

Perceived severity and susceptibility are the two constructs of the Health Belief Model, an intrapersonal behavior change theory designed to elucidate how beliefs predict commitment in health-protective behaviors and screenings. Perceived severity is the belief in the degree of harm from an acquired disease or harmful state as a result of a particular behavior. While perceived susceptibility is the subjective belief that a person may acquire a disease or enter a dire state due to a particular behavior [1]. The combination of perceived severity and perceived susceptibility is called a perceived threat. It has high relevance to many health behaviors [2]. The perceived threat is a determinant of the engagement in protective health behaviors, such as receiving protective health screenings [3]. Protective health behavior changes will only transpire if the disease is perceived as a threat and alongside the perception that engagement in the protective behavior will deter the risk of disease [4]. 

Leptospirosis is a new public health concern in Malaysia with an incidence rate of 30.2 per 100,000 in 2015 [5]. Despite the government efforts undertaken during the past several decades to increase the awareness regarding zoonotic diseases in the population, public understanding of this disease does not appear to have improved [6]. Previous studies in Malaysia found low to average knowledge, attitude and practice scores on leptospirosis [7,8,9,10,11,12]. Misconception about the disease is also widespread [13]. A study on the awareness of zoonotic diseases among rural workers found leptospirosis was among the least known compared to trichinosis, rabies, scabies, brucellosis, tuberculosis, and anthrax [14].

Although the importance of perceived threat in predicting health behaviors has been well studied in many disease contexts [15,16,17,18,19] less is known about public perceived severity and susceptibility of leptospirosis. However, perceptions of severity are highly subjective. Where some individuals will see a health problem as life-threatening and are ready to take precautionary action, others will see themselves as immune and preventive measures are unnecessary. Having good knowledge and a highly positive attitude score alone is not sufficient to translate into positive behavioral practice [9]. To fill this gap, we believe that a qualitative investigation is warranted to explore the perceived threat towards leptospirosis and how these constructs were conceived. The evidence from this study might potentially be of interest to many countries affected by leptospirosis. Ultimately, findings from this qualitative study may also be useful in identifying the best methodological designs that may assist in the improvement and development of an appropriate leptospirosis educational program. 

## 2. Materials and Methods

### 2.1. Study Settings

The study was conducted in Selangor and Kelantan states in Malaysia. Both states are facing rapid urbanization and waste management issues, leading to the proliferation of rodents and leptospirosis infection. These two states recorded the highest cases of leptospirosis in Malaysia [20]. Selangor is Malaysia’s most developed state, located strategically around the capital city, Kuala Lumpur. With its 5.79 million population, Selangor enjoys a highly developed infrastructure for major industry clusters and is a well-established investment haven backed by excellent state government support and an advanced commercial ecosystem. On the other hand, the Kelantan state is situated in the northeast region of peninsular Malaysia, with an estimated population of 1.54 million people. Kelantan is generally acknowledged as the most conservative and traditional state in Malaysia.

### 2.2. Study Design

The study used the phenomenology approach. We conducted focus group discussions (FGDs) because it provides content-rich information and evokes information that relates to personal reasons. It encourages group members to respond or behave in unanticipated ways, providing more opportunity for intuitive research [21].

### 2.3. Study Sample 

It is recommended that two and five focus groups were assigned to each category, depending on the complexity of the research question and the group composition. However, the inclusion of a few more than the suggested number of groups is recommended to account for any unforeseen confounders [22]. We assigned two groups for each state; rural and urban. We used purposive sampling and included respondents who were fluent in the Malay language. A total of 72 respondents were recruited; 38 in Selangor and 34 from Kelantan. We distributed invitations to participate in the study through emails. We asked the help of community leaders to invite respondents from the rural districts in both states. Those who responded were contacted and assigned a date and venue for the FGD.

### 2.4. Data Collection

We conducted FGD sessions at the research institution and houses of the community leaders. There were between eight to ten participants for each group, facilitated by two moderators. An interview guide comprised questions that explored the public perception of leptospirosis relating to the disease severity and perceived magnitude of risk. As some respondents were unable to describe the severity of leptospirosis, we asked respondents to rate their perceived severity of leptospirosis on a scale from one to ten. Even though this was rarely done in a qualitative inquiry, we felt the respondents needed some forms of objectivity to assist them in their responses. The FGDs were audio-recorded, and the duration ranged between 30–45 min. We transcribed the interviews in the Malay language. Field notes and audit trails were recorded and included in the data analysis. Data collection ceased after the eighth FGD when data saturation was achieved (no new information emerged). Ethical approval for this study was obtained from the Human Research Ethics Committee of the Universiti Sains Malaysia (USM/JEPeM/15120552) and the National Medical Research Registry. We obtained informed written consent from all the study participants.

### 2.5. Data Analysis

We used content analysis to generate category/categories as there was a lack of meaningful essence to generate themes. In a priori coding, categories were established before the analysis based upon the perceived severity (severe vs not severe) and susceptibility (highly susceptible vs. low susceptibility) of the Health Belief Model [23]. A category is a collection of similar data grouped together to enable researchers to identify and label the characteristics of the category. Categories are compared and contrasted with other categories, or if broad in scope, to be divided into smaller categories, and its parts identified and described [24]. During the analysis, similar chunks of text were identified, coded, explicated, and placed proximally according to the pre-set categories. If a category becomes too large, it may be separated into smaller units or subcategories. Professional colleagues agreed on the sub-categories, and coding was applied to the data. Necessary revisions were made, and the sub-categories were finalized to maximize mutual exclusivity and exhaustiveness.

## 3. Results

There were eight FGD sessions. The age of the study respondents ranged from 18 to 79 years old. The respondents were a mix of blue- and white-collar workers, self-employed, retirees and homemakers.

### 3.1. Perceived Severity of Leptospirosis

**Figure 1:**
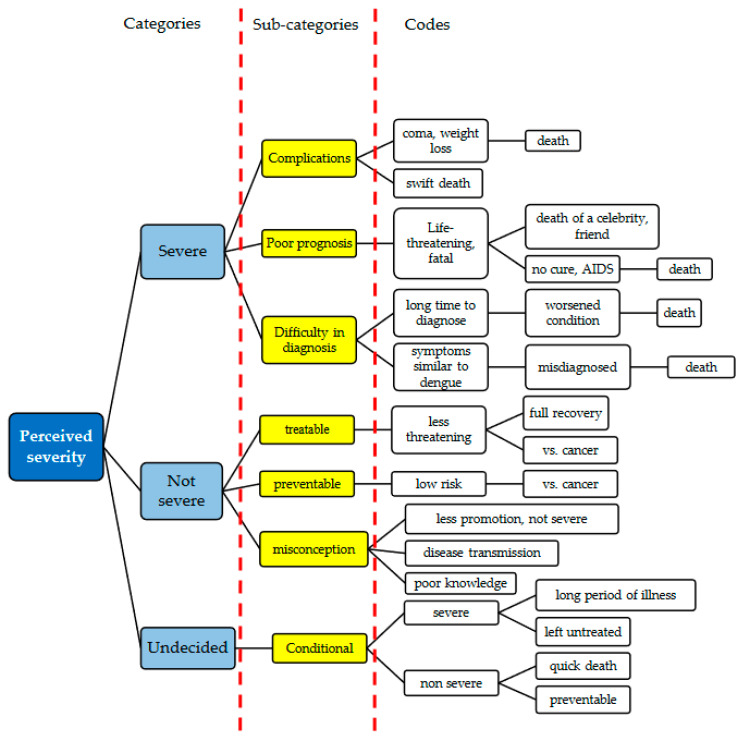
Mapping of the categories, sub-categories and codes pertaining to the perceived severity on leptospirosis infection.

Figure 1 demonstrates the mapping of codes and sub-categories belonging to the pre-set categories of “severe” and “not severe”. However, a third category emerged during the analysis; “undecided”.

In the study, respondents equated “severe” with “death”, “life-threatening’ or the frightening speed a person’s health could deteriorate or die.

“*It is severe because people could die from it. It leads to death, so it is just as dangerous as AIDS. The rat urine disease is categorized as one of the dangerous diseases. The father of my office mate; Linda, died from this disease within a week*” (urban)

For some respondents, their perceived severity was reinforced by the vivid memory of leptospirosis patients who died or had experienced severe consequences,

“*This disease can lead to death or comatose. I have seen a patient at the hospital who went from being overweight to extremely skinny*” (rural)

One respondent referenced the death of a celebrity that had gone viral in the social media as an indication of the disease fatality.

“*The death of an artist from leptospirosis went viral once. It is indeed dangerous*” (urban)

The perception of severity was also based on the difficulty in diagnosing the disease.

“*It takes a very long time to confirm whether it is leptospirosis. One must go to the hospital for a blood test, then monitor the progress, watch for symptoms at home, and wait for the laboratory results. The detection occurs quite late, and meanwhile, patient health worsens. So yes, it can lead to death*” (urban)

Pertaining to the perception of severity of leptospirosis on a scale of one to ten, some respondents commented that leptospirosis was “not a severe disease” because it is treatable and preventable, making it a less threatening disease.

“*I give a score of 2 because we know how to prevent it and thus can be more careful about it*” (urban)

“*I give it a score of 5. Leptospirosis is 80% treatable*” (urban)

However, several respondents based their perception of severity on the comparison of leptospirosis with cancer,

“*I give it a score of 5. It is not that dangerous. I mean, compared to cancer, it is not that serious*” (urban)

“*It is not like cancer that can suddenly appear in our body without us knowing if or when we will get it. But leptospirosis can be prevented right from the beginning*” (urban)

One respondent gave a low score based on the absence of leptospirosis health campaigns by the Ministry of Health.

“*If it is not dangerous, there would not be any campaigns to remind the public. However, if it is dangerous, campaigns would be in place. The Ministry of Health does not seem to highlight leptospirosis much*” (rural)

Perceived severity was also based on the misconception regarding leptospirosis and the lack of knowledge regarding the disease,

“*I think leptospirosis is the most dangerous disease because people can die from it and because the parasite comes from the mosquitoes that are present everywhere (in the bushes, woods, and house). It can bite animals like rats that can bite us, humans. So, if we get a fever, we might have leptospirosis*” (urban)

“*My score on the fear scale is 5. I cannot give a ten because I do not know much about the disease [laugh]*” (rural)

A third category emerged during the analysis i.e. “undecided”. Among those who were undecided, their responses demonstrated a “conditional” perception of severity indicated by the frequent use of the word “if”,

“*If a person dies straight away, then it is not dangerous. But if a person remains ill for a long time, it is scarier*” (rural)

“*I give a score of 5–6 if it is preventable. But if one does not seek any treatment for it, then I give it a 10 in terms of severity*” (urban)

### 3.2. Perceived Susceptibility towards Leptospirosis

**Figure 2:**
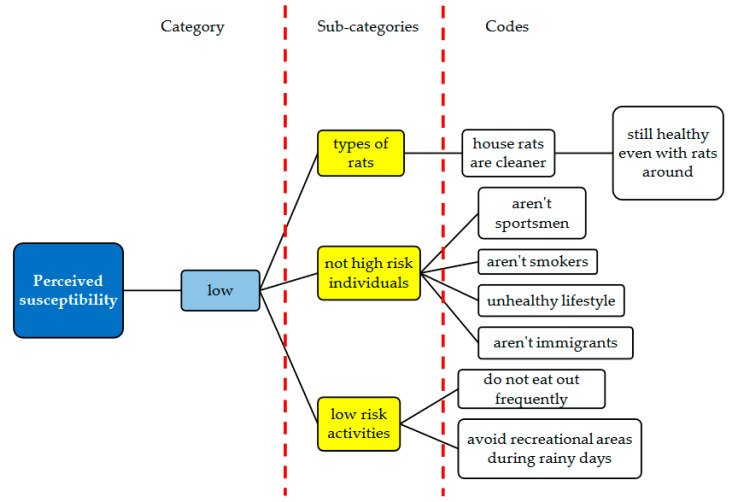
Mapping of the category, sub-categories and codes pertaining to the perceived susceptibility of leptospirosis infection.

Mapping of the responses showed that all respondents had a low perception of susceptibility to leptospirosis (Figure 2). Hence there was only one category that emerged under the perceived susceptibility of leptospirosis.

The type of rats seemed to influence respondents’ perception of susceptibility. House rats were considered “cleaner” and “safer”, thus did not pose any risk of transmitting leptospirosis,

“*The disease is only present in the marketplace. I have rats in my house that urinate everywhere; however, it has been three years, and I’m still fine, which means that the urine of house rats has no germs and is not dangerous*” (rural)

Respondents also believed they were not susceptible to leptospirosis because they were not high-risk individuals and did not participate in high-risk activities that may lead to leptospirosis infection. The common perception was that only a specific group of people were at risk of the disease, particularly those whose lifestyles made them more susceptible to leptospirosis,

“*To my knowledge, sports enthusiasts were the ones commonly infected by this disease*” (urban)

“*In my opinion, people who are at risk of this disease are those who practiced unhealthy lifestyle such as smoking and preferred to eat outside where food hygiene is not guaranteed. We must also be careful with the selection of the recreational areas that we visited to prevent exposure to infection. Pay attention to the media and should be aware of the places and avoid going to these places especially when it rains*” (rural)

They also had a biased belief that only immigrant workers were more susceptible to leptospirosis,

“*I believe that the immigrants are more prone to leptospirosis infection because they eat and simply throw away their food waste; this can lead to rat infestation*” (urban)

However only one outlier comment was observed,

“*I regularly wash my hands, but the garbage disposal is poorly managed, and there are house pests everywhere. The children, they love to play in water puddles when it rains. All these are very risky, and we are all at risk*” (urban)

## 4. Discussion

The perceived severity of leptospirosis infection did not vary between rural and urban respondents; there were mixed responses from low to highly severe. In contrast to findings from Allwood et al., urban households without a history of leptospirosis had a higher perception of the severity of leptospirosis [25]. Similarly in another study of the perceived threat of type-2 diabetes, rural adults’ perceived threat was relatively low, in comparison to their actual threat, i.e., body mass index and family history [26]. 

The majority of the respondents in our study perceived leptospirosis as severe and highly severe, based on the poor disease prognosis, lack of a cure, rapid deterioration rate, and most importantly, mortality risk. This finding is consistent with Rosenstock [1] who postulated that a person may perceive a health problem in terms of its clinical consequences and whether it could lead to death. It seemed that death was considered synonymous with leptospirosis, owing to the reports made viral by mass and social media in Malaysia. The reports of deaths due to leptospirosis were often broadcasted during prime-time news. Media coverage on leptospirosis in Malaysia also tend to highlight high-profile cases that were novel and dramatic, creating a situation known as the “social amplification of risk” [27]. Information provided by the media may lead to over- or under-estimation of the disease severity [28]. In an investigation by Young et al., participants considered diseases that are reported frequently by the media as more serious and severe and as having a higher disease status than those reported less commonly [29]. Consistent with this, Frost, Frank, and Maibach found a weak association between the frequency of reporting by the print media and the actual risk and mortality rates [30]. However, concurrent presentations of objective information about the diseases can mitigate this effect; this explained the fact that those who were knowledgeable about leptospirosis did not rate the disease as severe. 

Further, some respondents rated leptospirosis as severe because of its challenging diagnosis. The similarity of its symptoms to those of dengue fever might lead to late diagnosis and treatment. This reason behind the belief of disease severity has merit because several symptoms of leptospirosis closely resemble those of dengue that is endemic to Malaysia, with the incidence rate of 328.3 cases per 100,000 populations [31].

Perception of illness can influence patients’ emotional response to the illness and their coping behaviors, including adherence to treatment [32]. For instance, perceived objective severity had a preventive effect on the risk of brushing less than twice a day among young adolescents [33]. However, according to Janz and Becker [34] perceived severity has a weak association with preventive health behaviors as compared to other Health Belief Model dimensions. It was speculated that these findings might be partly attributed to the challenges faced by the study respondents in conceptualizing this dimension such as: (1) when they are asymptomatic; (2) for health threats that are usually considered long term; and (3) concerning medical conditions with which they have had little or no personal experience [34]. Our study findings confirmed all of the above postulations.

Perceived susceptibility is a stronger contributor to the understanding of preventive health behavior [34]. However, our study respondents believed they were not susceptible to leptospirosis infection. Fear and worries are essential determinants of high perceived risk; in a study of parents’ perceptions of diseases and decisions about vaccinations, immunizers dreaded the outcomes of the diseases, especially those in which they were unaccustomed. This fear motivated them to take the risk of immunizing their children [35]. Comparative ratings of worry were more consistent with the population prevalence of disease. Perceived risk was significantly higher for cancers than for other diseases [36,37,38]. It has been demonstrated that elevated perceptions of risk for one disease correspond to lower worry about other diseases thereby potentially compromising the likelihood that individuals will engage in protective actions to reduce their risk for these other diseases [39]. Furthermore, leptospirosis is not easily diagnosed; therefore the real burden of the disease and its susceptibility can be underestimated by the public [40].

Low perceived susceptibility towards leptospirosis infection may also be due to the poor knowledge about the disease. In a study of rural and urban respondents in Selangor, 57–80.3% had poor knowledge about leptospirosis and its prevention [7,8]. It may also be influenced by the belief towards the disease. Pathman et al. reported 40% of their study respondents had the belief that leptospirosis was not dangerous and that they were not at risk of contracting the disease, believing only elderly and people who are immunocompromised were at a higher risk [9].

### Limitations

Some biases may be introduced by the moderator effect during the FGD sessions. However, there were only slight variances between the groups, suggesting minimal bias. Efforts to address stability and consistency in the research include having multiple researchers, group debriefings, and proper records of audit trails and field notes. Respondents were predominantly Malays ethnic; thus interpretation and generalizability of these findings may be limited. Future research should examine differences in the perceptions across racial/ethnic and individuals who are at higher risk for leptospirosis infection. Future studies are needed to examine perceived barriers, the interrelationships among individuals’ perceptions for various conditions as well as the implications of modifying these perceptions on both behavioral and health outcomes. 

## 5. Conclusions

Leptospirosis prevention in endemic areas is highly dependent on health education initiatives developed following the public perception of the disease. The present study revealed an interesting pattern in the perception of severity. Although some respondents perceived leptospirosis as severe, it may not be enough to influence their preventive health behavior, suggesting perceived susceptibility should instead be the main focus. Poor knowledge contributed to the low perceived threat of leptospirosis infection, indicating the importance to raise awareness about the disease to encourage preventive lifestyle behaviors.
